# Optical genome mapping: Unraveling complex variations and enabling precise diagnosis in dystrophinopathy

**DOI:** 10.1002/acn3.52245

**Published:** 2024-11-22

**Authors:** Jiahui Mai, Jing Duan, Xiaoyu Chen, Liqin Liu, Dachao Liang, Tao Fu, Gang Lu, Wai Yee Chan, Xufeng Luo, Feiqiu Wen, Jianxiang Liao, Zhuo Li, Xinguo Lu

**Affiliations:** ^1^ Department of Neurology Shenzhen Children's Hospital of China Medical University No. 7019 Yitian Road, Futian District Shenzhen 518038 Guangdong PR China; ^2^ Department of Neurology Shenzhen Children's Hospital No. 7019 Yitian Road, Futian District Shenzhen 518038 Guangdong PR China; ^3^ Shenzhen A‐Smart Medical Research Center, Room 516 Shenzhen Research Institute of the Chinese University of Hong Kong 10, 2nd Yuexing Road, Nanshan District Shenzhen 518000 Guangdong China; ^4^ The Chinese University of Hong Kong‐Shandong University (CUHK‐SDU) Joint Laboratory on Reproductive Genetics School of Biomedical Sciences, The Chinese University of Hong Kong Hong Kong Hong Kong

## Abstract

**Objective:**

Approximately 7% of individuals with dystrophinopathy remain undiagnosed at the genetic level using conventional genetic tests like multiplex ligation‐dependent probe amplification (MLPA) and next‐generation sequencing (NGS). We used the optical genome mapping (OGM) technology to detect and analyze uncommon mutations or structural variations (SVs) within the *DMD* gene, thus contributing to more precise clinical diagnoses.

**Methods:**

We herein included eight patients with dystrophinopathy (six males and two females) in whom pathogenic variants of the *DMD* gene could not be accurately identified using MLPA and NGS. Clinical data were collected for all patients and genetic testing was performed using OGM.

**Results:**

Conventional methods (MLPA and NGS) failed to detect pathogenic mutations in six out of eight individuals (four males and two females). OGM testing uncovered rare mutations in the *DMD* gene in four patients, including a pericentric inversion in chromosome X (one male), a complex rearrangement (one male), and two X–autosome translocations (two females). No mutations were detected in the remaining two male patients. OGM also accurately mapped balanced X–autosome translocations in female patients, defining chromosomal breakpoints. In the other two male patients in whom MLPA suggested non‐contiguous exon duplications or deletions in the *DMD* gene, OGM characterized one case as a complex rearrangement and the other as a deletion within the *DMD* gene.

**Interpretation:**

OGM is a valuable diagnostic tool for dystrophinopathy patients with negative results from conventional genetic tests. It can effectively elucidate complex SVs and pinpoint breakpoints in X–autosomal translocations in female patients, facilitating prompt and appropriate interventions.

## Introduction

Dystrophinopathy, which includes Duchenne muscular dystrophy (DMD, MIM 310200) and Becker muscular dystrophy (BMD, MIM 300376), is an X‐linked recessive neuromuscular disorder that occurs from mutations in the *DMD* gene and its prevalence ranges from of 15.9 to 19.5 per 100,000 live male births.^1^ DMD is characterized by progressive, symmetrical muscle weakness that predominantly affects proximal muscles. Patients with DMD usually die from respiratory or heart failure in their twenties. As DMD has X‐linked recessive inheritance, it usually only affects hemizygous males, and the majority of female carriers remain asymptomatic.^2^

The *DMD* gene is located at the Xp21 locus and spans more than 2 Mb. It contains 79 relatively small exons (32–269 bp). Exon deletions are the most common pathogenic variants (50%–70%) in dystrophinopathy, and 30% of patients have point mutations and ~ 5% have exon duplications.^3^, ^4^ In some of the recent studies, the disease‐causing mutation could not be identified in a small proportion of patients in the clinic (2%–7%).^5^, ^6^, ^7^ It is possible that underlying some of these cases are complex structural variants (SVs) that are not detectable by current clinical genetic testing methods, such as multiplex ligation‐dependent probe amplification (MLPA), Sanger sequencing, and next‐generation sequencing (NGS).^1^, ^6^, ^8^, ^9^

Recent advances in DNA sequencing and genome mapping have revolutionized the fields of genomic science and genomic medicine. Two novel methods, namely long‐read sequencing and optical genome mapping (OGM), can identify complex mutations and SVs.^10^, ^11^ OGM is a cytogenomic technology that uses restriction enzyme mapping of single DNA molecules to generate detailed, ordered whole‐genome restriction enzyme maps. It can recognize all types of genomic SVs, such as inversions, balanced translocations, and genomic imbalances (insertions and deletions).^11^, ^12^ OGM can also reportedly locate intricate variants in some DMD cases.^13^, ^14^

As early as 2017, Barseghyan et al. in their research used OGM to conduct genetic testing on six patients with confirmed *DMD* gene SV mutations and three carrier mothers, demonstrating the ability of OGM to detect large deletions, insertions, and inversions in both hemizygous and heterozygous states of the *DMD* gene.^14^ Diverging from this approach, our investigation used OGM for the genetic analysis of eight dystrophinopathy patients who encountered diagnostic hurdles post‐MLPA and NGS, with the objective of conclusively determining the disease‐causing genetic variations. The OGM assessments successfully delineated the specific mutation sites within the *DMD* gene in six of these cases.

## Materials and Methods

### Patients

The research was approved by the Ethics Committee of Shenzhen Children's Hospital and it involved eight participants diagnosed with dystrophinopathy. Patient inclusion criteria were as follows: (i) elevated serum creatine kinase (CK) levels; (ii) male patients with calf muscle hypertrophy, symmetrical limb weakness, and delayed motor development, which are consistent with the clinical presentation of dystrophinopathy or those with muscle biopsy results showing the absence of or reduced dystrophin protein expression; (iii) female patients with muscle biopsy results showing the absence of or reduced dystrophin protein expression; and (iv) no significant pathogenic variants detected in the *DMD* gene or other genes through MLPA and NGS or MLPA/NGS findings suggestive of structural variations in the *DMD* gene, such as non‐contiguous exon deletions or duplications. The exclusion criterion was a history of bone marrow stem cell transplantation or blood transfusion within the previous year. After obtaining informed consent, all participants, and when feasible, their mothers, underwent peripheral blood sample collection.

### Optical genome mapping (OGM)

Ultra‐high‐molecular weight (UHMW) gDNA was isolated from 400 μL of peripheral blood using the SP Blood and Cell Culture DNA Isolation Kit from Bionano Genomics (San Diego, CA, USA) as per the Bionano Prep® SP Frozen Human Blood DNA Isolation Protocol v2 (document number: 30395). Subsequently, 750 ng of the isolated UHMW gDNA was fluorescently tagged with the Bionano Prep DLS Kit (Bionano Genomics, San Diego, CA, USA) according to the Bionano Prep Direct Label and Stain (DLS) Protocol (document number: 30206). Then, the labeled UHMW gDNA was loaded onto a Saphyr chip and linearized, followed by imaging on the Saphyr instrument (Bionano, San Diego, USA). These data were further processed by the Bionano Solve software version 3.7 (Bionano, San Diego, USA) using the *de novo* assembly and variant annotation. Data visualization was processed using the Bionano Access software version 1.7 (Bionano, San Diego, USA). All procedures for OGM were conducted in Shenzhen A‐Smart Medical Research Center.

### Sanger sequencing

The Magnetic Blood Genomic DNA Kit (Tiangen Biotech, Beijing, China) was used to obtain genomic DNA from peripheral blood and a fragment encompassing exon 42 of *DMD* gene was amplified using the primers 5′‐TGTTCTGGCACTATGAATGA‐3′ and 5′‐ AACTTAATGGAGGAGGTTTC‐3′. Thereafter, the PCR product was validated by Sanger sequencing.

## Results

### Patients and summary of the findings

To identify pathogenic variants and elucidate suspected complex SVs, we performed OGM for eight individuals (two females and six males; age, 1–23 years) diagnosed with dystrophinopathy and one biological mother. Among them, six patients had previously undergone routine clinical NGS of the *DMD* gene, yielding negative results. One of the remaining two patients showed microduplications of exons 45–47 and 52–60 of the *DMD* gene using MLPA, whereas the other showed deletions of exons 42 and 45–50 of the *DMD* gene using MLPA, indicating potential complex SVs (Table 1).

**Table 1:** Relevant clinical and genetic findings of patients.

Case ID	1	2	3	4	5	6	7	8
Sex	M	M	M	M	F	F	M	M
Age at diagnosis	5 years and 3 months	8 years	6 years	1 year	1 years and 2 months	10 years and 7 months	3 years	6 years and 4 months
Family history	No	Maternal cousin had a similar medical history and died at ~30 years of age	Elder brother diagnosed with DMD	Patient's uncle and cousin both have DMD, and the uncle died young.	No	No	No	No
Clinical features	Could crawl at 7 months, walk at 1 year, and run and jump at the same time as peers	Could walk at 15 months, unsteady gait, poor running and jumping at 3 years; difficulty climbing stairs and squatting at 5 years; enlarged calf muscles, spinal scoliosis, and Gower's sign identified during the first visit to our hospital	Could walk at 16 months, poor running and jumping at 3 years; difficulty squatting at 6 years; enlarged calf muscles and Gower's sign identified during the first visit to our hospital	Could walk at 17 months, difficulty climbing stairs and squatting at 4 years; Gower's sign at 4 years; enlarged calf muscles identified during the first visit to our hospital	Could stand at 11 months, could walk a few steps independently at 14 months	Poor head control at 3 months; could walk at 16 months; uncoordinated movements at 4 years; frequent falls at 5 years; difficulty climbing stairs and squatting at 10 years	Slow climbing stairs at 5 years; enlarged calf muscles and mild Achilles tendon contracture identified during the first visit to our hospital	Poor running and jumping at 4 years; enlarged calf muscles identified during the first visit to our hospital
CK level (IU/L)	7000‐33568	3000‐39336	10126	20000	2310‐9597	58340	21412‐30541	11740
Muscle biopsy	Absence of dystrophin expression	Not done	Not done	Not done	Dystrophin reduction	Dystrophin reduction	Absence of dystrophin expression	Absence of dystrophin expression
Karyotype	Negative	Not done	Not done	Not done	46,X,der(X)ins (X;8) (p21;q23q22)	46,X,t(X;5) (p11.4;p15.1)	Not done	Not done
MLPA	Negative	Negative	Exons 45–47 and exons 52–60 duplication	Exon 42 and exons 45–50 deletion	Negative	Negative	Negative	Negative
NGS	Negative	Negative	Not done	Not done	Negative	Negative	Negative	Negative
OGM	ogm[GRCh37] inv(X) (p21.1; q26.2)	ogm[GRCh37] dup(X) (p21.1p21.1) and ogm[GRCh37] fus(X: X) (p22.11; p21.1)	ogm[GRCh37] dup inv(23) (p21.1; p21.1)	ogm[GRCh37] Xp21.1(31825585_32077899)x0	ogm[GRCh37] t(X; 8) (p21.1; q22.1)	ogm[GRCh37] t(X; 5) (p21.1; p15.33)	Negative	Negative

CK, creatine kinase; DMD, Duchenne muscular dystrophy; F, female; M, male; MLPA, multiplex ligation‐dependent probe amplification; NGS, next‐generation sequencing; OGM, optical genome mapping.

OGM revealed pathogenic variants in 6/8 cases, including an inversion, a deletion in the *DMD* gene, each found in one male patient, complex rearrangements in two male patients, and translocations between the X chromosome and autosomal chromosome in two female patients.

### Identification of rare SVs in male DMD cases concluding the diagnostic odyssey

**Figure 1:**
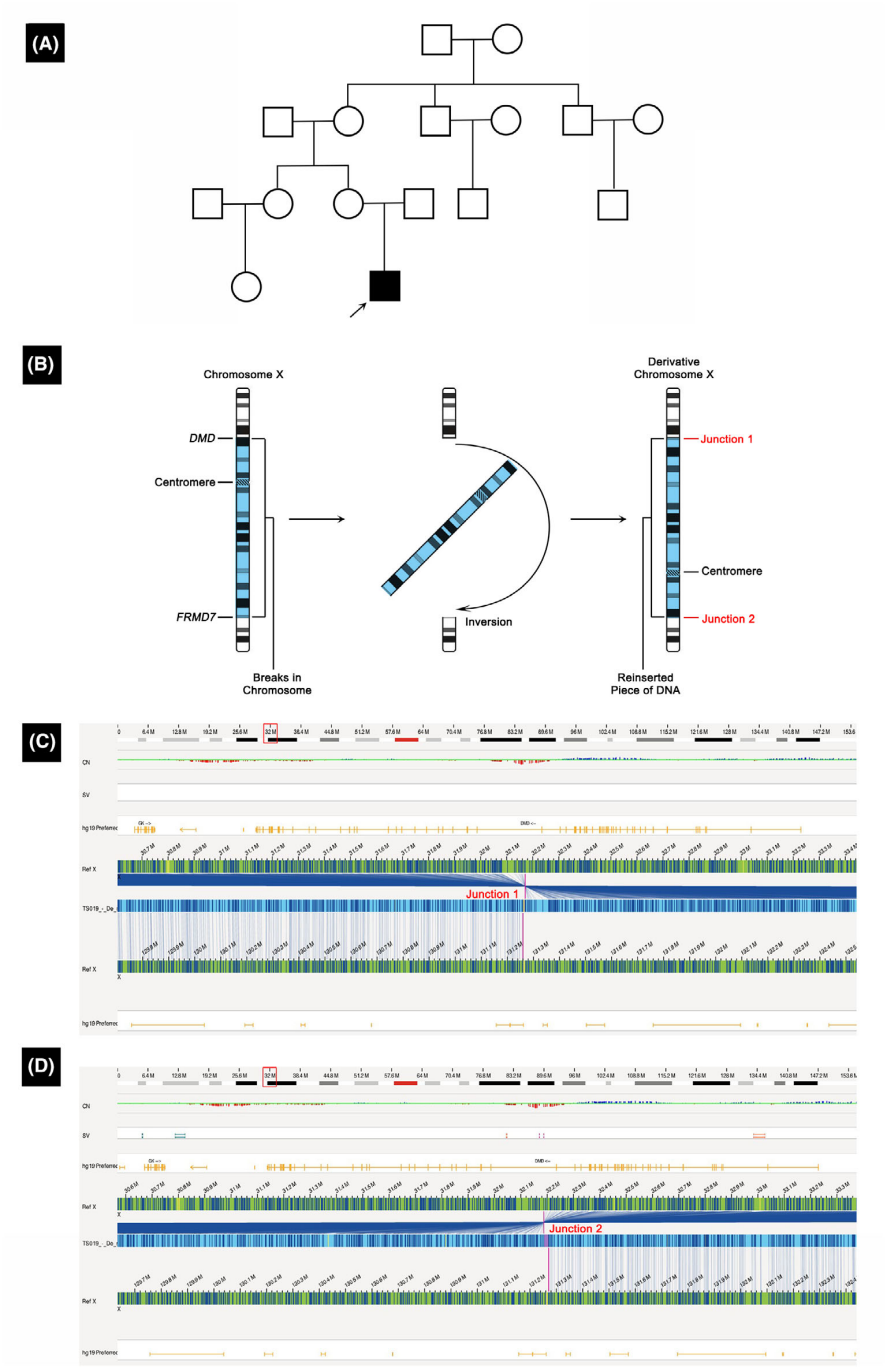
Characterization of inversion in Case 1. (A) The family pedigree of Case 1. There is no history of neuromuscular diseases. (B) A schematic of the inversion. The breakpoints are located in *DMD* and *FRMD7*. (C) Junction 1 is near the enzyme mapping sites located at 32170892 and 131260066. (D) Junction 2 is near the enzyme mapping sites located at 32187162 and 131270897.

Case 1 was of a 6‐year‐old boy born to healthy, non‐consanguineous Chinese parents with no history of neuromuscular diseases (Table 1, Fig. 1A). At 3 years of age, he showed elevated muscle enzymes levels. MLPA and NGS of the *DMD* gene did not detect any mutations. A muscle biopsy performed at 5 years and 3 months of age showed no dystrophin expression, resulting in a pathological diagnosis of DMD. His OGM analysis revealed a 99‐Mb pericentric inversion, ogm[GRCh37] inv(X) (p21.1q26.2), which was disrupting the coding sequence of the *DMD* gene, with breakpoints located in intron 44 of the *DMD* gene and downstream of the *FRMD7* gene (Fig. 1B–D). Subsequent karyotyping efforts could not validate the inversion because of the high similarity of the bands in the karyotype before and after the inversion.

**Figure 2:**
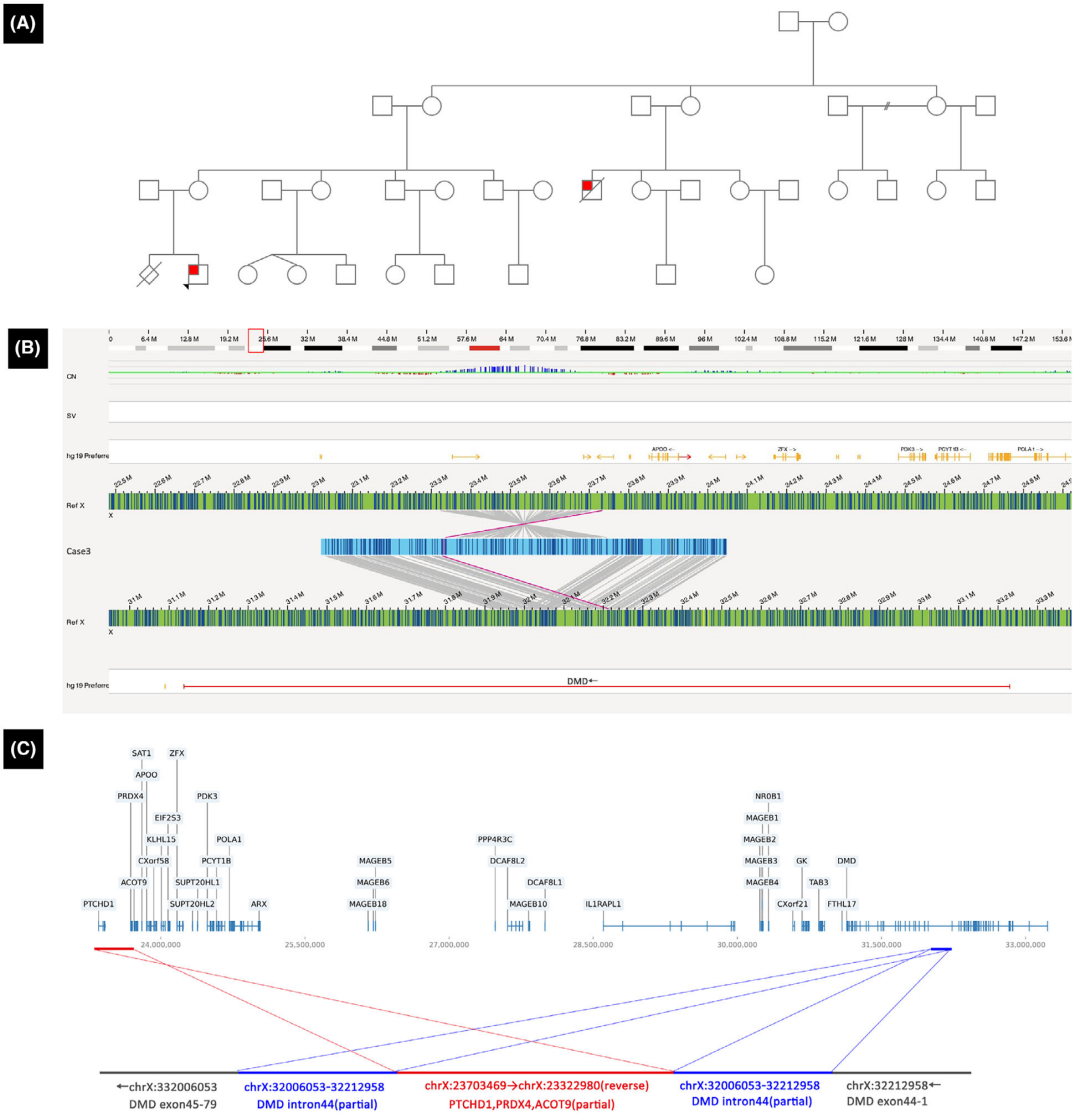
Characterization of complex rearrangement in Case 2. (A) The maternal cousin of the patient shown as Case 2, as indicated in the family pedigree, had a similar medical history and died at ~30 years of age. (B) Genome‐browser view of OGM results showing the presence of duplications in specific chromosomal regions, including chrX:320006053‐32212958 and chrX:23322980‐23703469. In addition, it shows the inversion and insertion of the chrX:23322980‐23703469 duplication into the two copies of chrX:320006053‐32212958. (C) A schematic of the complex rearrangement.

Case 2 was of an 8‐year‐old boy born to healthy non‐consanguineous Chinese parents (Table 1, Fig. 2A). He showed elevated muscle enzymes 2 days after birth. By the age of 3 years, he showed unstable gait and impaired running and jumping abilities. His maternal cousin had a similar medical history and had died at ~30 years of age. Both NGS and MLPA tests yielded negative results. OGM analysis of this boy and his mother indicated the presence of duplications in specific chromosomal regions, including chrX:320006053‐32212958 (a partial segment of *DMD* intron 44) and chrX:23322980‐23703469 (encompassing *PTHD1*, *PRDX4*, and a partial segment of *ACOT9* gene). Furthermore, it revealed the inversion and insertion of the chrX:23322980‐23703469 duplication into the two copies of chrX:320006053‐32212958 (Fig. 2B,C). This complex SV was inherited from his mother.

### Identification of the breakpoint of balanced autosome–X translocations in female dystrophinopathy patients

**Figure 3:**
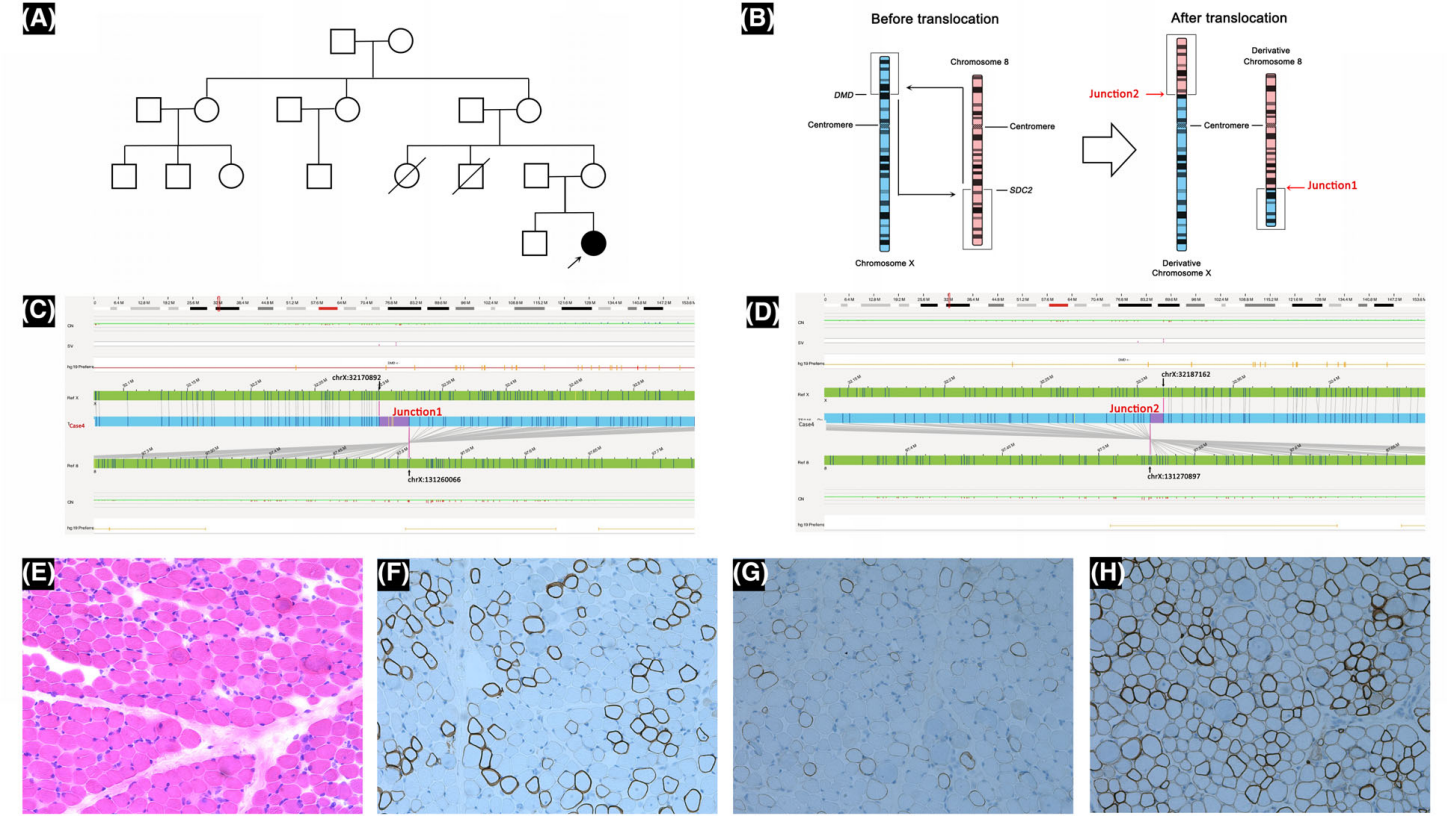
Characterization of translocation in Case 5. (A) The family pedigree of Case 5 shows no history of neuromuscular diseases. (B) A schematic of the translocation showing that the breakpoints are located in *DMD* and *SOC2*. (C) Junction 1 is near the enzyme mapping sites located at chrX:32170892 and chrX:131260066. (D) Junction 2 is near the enzyme mapping sites at chrX:32187162 and chrX:131270897. (E) HE staining of muscle biopsy revealed (i) a slight increase of connective tissue in the fascicles, (ii) muscle fibers with hypertrophy and atrophy, (iii) muscle fibers with necrosis and regeneration, and (iv) a few hypercontracted acidophilic muscle fibers (×200). Immunohistochemistry. Dystrophin staining of muscle biopsy showed a reduction in the expression of dystrophin‐C (F), dystrophin‐N (G), and dystrophin‐R (H).

Case 5 was of a 14‐month‐old girl conceived by *in vitro* fertilization, with no significant family history (Table 1, Fig. 3A). Elevated CK levels (2310–9507 IU/L) were observed from 2 months of age. At 14 months, she started walking independently for short distances. Muscle biopsies revealed partial absence of dystrophin expression (Fig. 3E–H), which is consistent with the pathological features of DMD carriers. Both NGS and MLPA tests yielded negative results. Chromosomal karyotyping suggested an 8q23q22 insertion in Xp21 chromosomes, particularly 46,X,der(X)ins (X;8) (p21;q23q22), or a translocation between chromosomes X and 8, 46,X,t(X;8) (p21;q22). OGM analysis confirmed a translocation between chromosomes X and 8, denoted as t(X;8) (p21.1;q22.1) (Fig. 3B–D). This translocation involved the breakpoint on chromosome X within the region chrX:32300372‐32313666, which included partial intron 42, exon 43, and partial intron 43 of the *DMD* gene. Furthermore, the breakpoint on chromosome 8 was located within intron 1 of the *SDC* gene.

**Figure 4:**
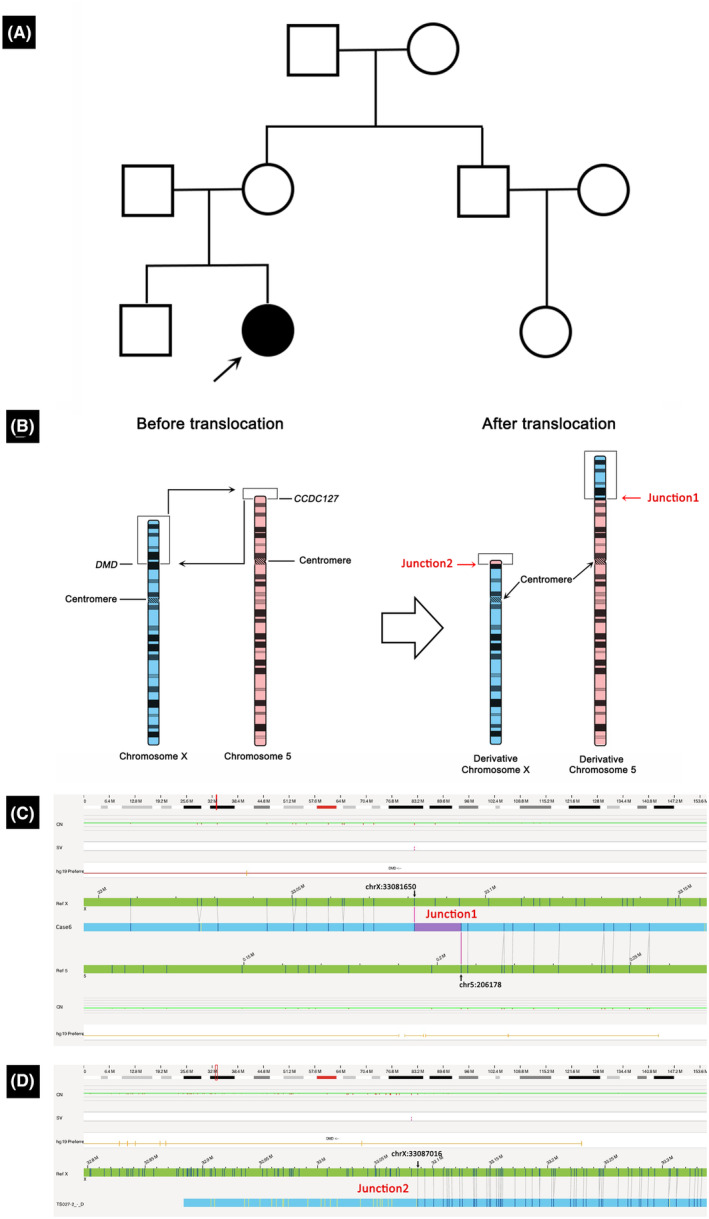
Characterization of translocation in Case 6. (A) The family pedigree of Case 6 shows no history of neuromuscular diseases. (B) A schematic of the translocation. The breakpoints are located in *DMD* and *CCDC127* genes. (C) Junction 1 is near the enzyme mapping sites located at chrX:33081650 and chr5:206178. (D) Junction 2 is near the enzyme mapping sites at chrX:33087016 and chrX:198556.

Case 6 was of a 23‐year‐old female patient with low muscle strength since childhood (Table 1). She started to walk independently at 16 months, indicating developmental delay. At 10 years of age, she had trouble climbing stairs and squatting and showed calf muscle hypertrophy. Muscle biopsies revealed partial absence of dystrophin expression, which is consistent with the pathological features of DMD carriers. However, clinical genetic tests (NGS and MLPA) yielded negative results. Chromosomal karyotyping suggested a translocation between chromosomes X and 5, t(X;5) (p11.4;p15.1). OGM analysis confirmed a translocation between chromosomes X and 5, t(X;5) (p21.1;p15.33), with breakpoints within intron 1 of the *DMD* gene and exon 3 of the *CCDC127* gene (Fig. 4).

### Validation of complex SVs suspected based on the MLPA method

**Figure 5:**
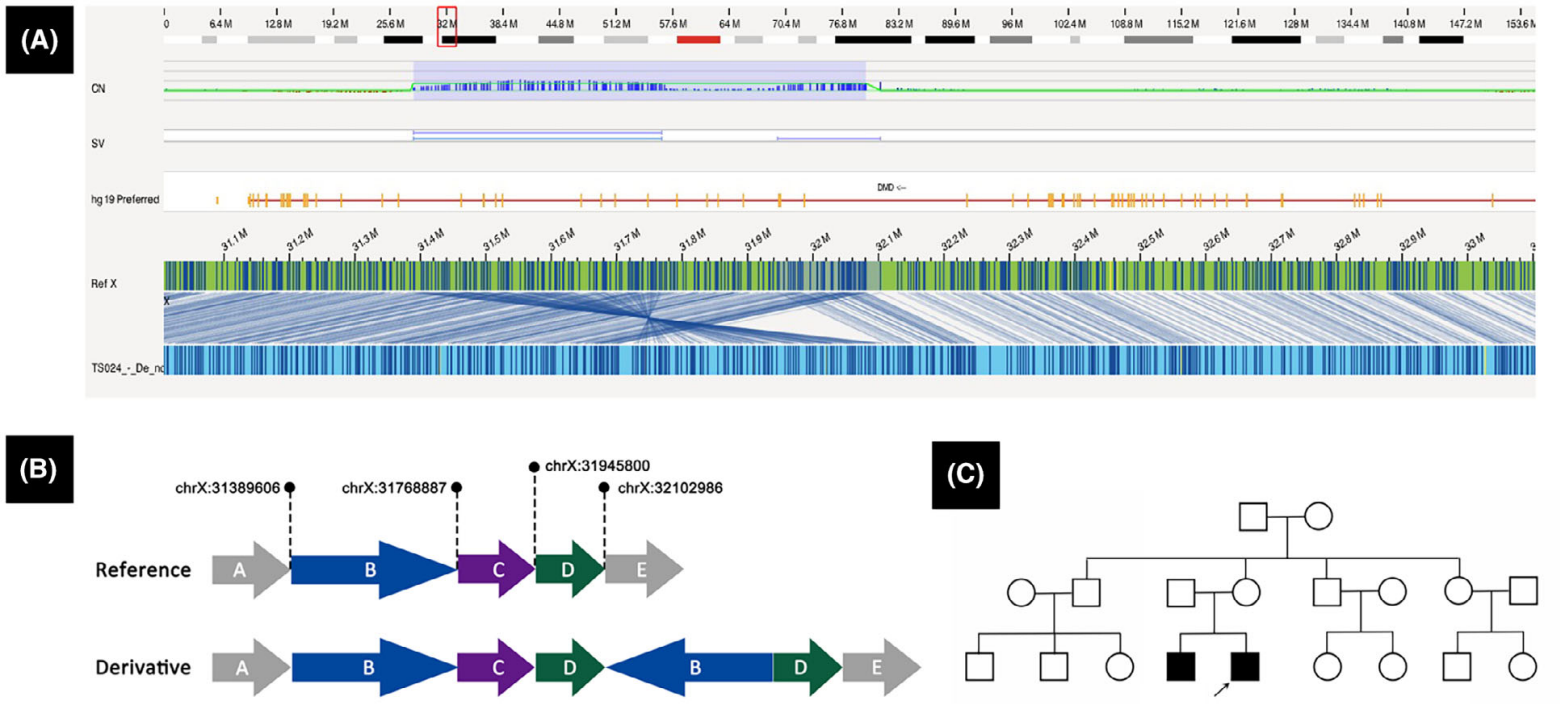
Characterization of complex rearrangement in Case 3. (A) Genome‐browser view of OGM results showing inverted microduplication of exons 52–60 and concatenated microduplication of exons 45–47, both inserted between exon 47 and exon 48. (B) A schematic of the complex rearrangement. (C) In the family pedigree of Case 3, his elder brother had a similar medical background.

Case 3 was of a 10‐year‐old boy; he showed significantly increased CK levels 2 months after birth, along with delayed development compared to age‐matched peers (Table 1). His elder brother had a similar medical background. The MLPA assay revealed two microduplications covering exons 45–47 and 52–60 of the *DMD* gene. The OGM results confirmed the MLPA findings and provided more details on the variant's structure, revealing an inverted microduplication of exons 52–60 and a concatenated microduplication of exons 45–47, both inserted between exon 47 and exon 48 (Fig. 5).

**Figure 6:**
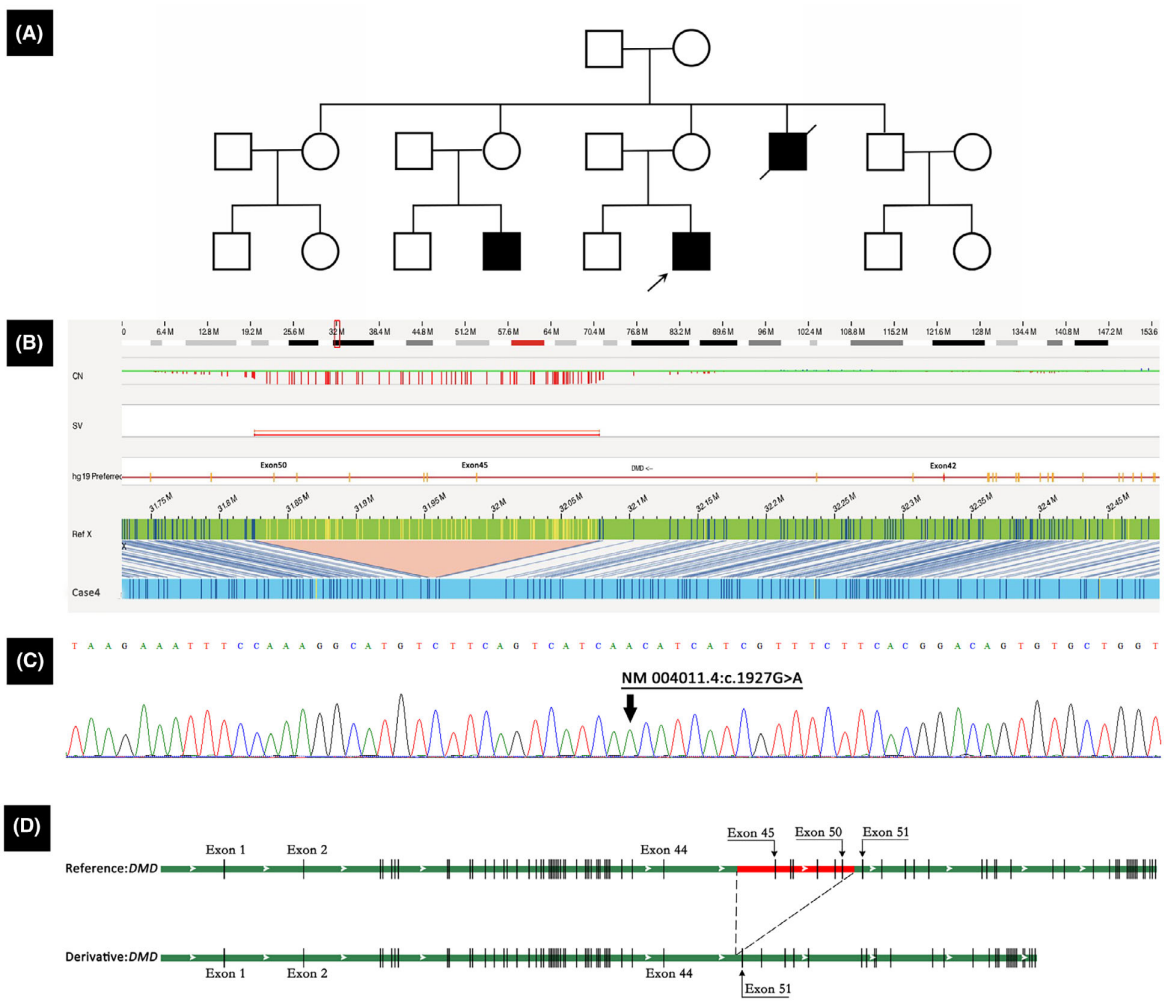
Characterization of deletion in Case 4. (A) In the family pedigree of Case 4, both his cousin and uncle had similar medical histories and were diagnosed with DMD. (B) Genome‐browser view of OGM results revealing the deletion spanning exons 45–50. (C) In exon 42, Sanger sequencing revealed a point mutation, NM_004011.4: c.1927G>A. (D) A schematic of the deletion. The breakpoints are located in the intron 44 and intron 50 of *DMD*.

Case 4 was of a 9‐year‐old boy; at 1 year of age, he showed significantly elevated CK levels, fluctuating at around 20,000 IU/L (Table 1). He started to walk independently at 17 months; however, later, at the age of 4 years, he had trouble with squatting and climbing stairs. Both his cousin and uncle had similar medical histories and were diagnosed as having DMD (Fig. 6A). MLPA testing revealed deletions of exons 42 and 45–50 of the *DMD* gene. However, OGM analysis indicated the absence of only exons 45–50 and not exon 42 (Fig. 6B). Further validation by Sanger sequencing verified the presence of exon 42 and found a point mutation, NM_004011.4:c.1927G>A, in the target sequence detected by the probe of exon 42, which caused a decreased probe signal and led to a false‐positive result for exon 42 deletion (Fig. 6C). The mutation was eventually identified as the deletion of exons 45–50 (Fig. 6D).

## Discussion

The clinical significance of determining the molecular cause underlying neuromuscular disorders, particularly dystrophinopathy, has increased over the last decade as several individualized and mutation‐specific therapeutic approaches have already been approved (e.g., viltolarsen for exon 53 skipping in DMD), with many others currently under investigation. Conventionally, *DMD* gene variants are identified using a synergistic approach combining MLPA and NGS.^8^, ^15^ Despite these advancements, a non‐negligible fraction of dystrophinopathy cases (~7%) remain genetically unsolved.^6^, ^7^ In such scenarios, the use of a combination of investigative modalities, including muscle biopsy, muscle mRNA analysis, full‐length sequencing of the *DMD* gene, and RNA sequencing, is advocated.^7^, ^16^, ^17^

In our investigation, we used OGM to precisely pinpoint gene variants in six patients with dystrophinopathy whose genetic diagnosis was previously found to be challenging with MLPA and NGS. In four patients, OGM analysis detected pericentric inversion of the X chromosome (*n* = 1), complex rearrangement (*n* = 1), and X–autosome translocations (*n* = 2). In addition, we found two cases with potential SVs suggested by MLPA results, one with a complex rearrangement and another with the deletion of the *DMD* gene. Our findings emphasize the significance of splice‐altering variants, such as SVs, as a prevalent cause of undiagnosed dystrophinopathy, thus positioning OGM as an efficacious tool for their detection.

In our study, we addressed the challenge of diagnosing dystrophinopathy in cases wherein clinical symptoms are indicative but genetic tests, such as MLPA and NGS, yield negative results. This discrepancy can lead to a misdiagnosis or delayed diagnosis, thus affecting treatment initiation and family planning decisions. Case 1 was initially misdiagnosed with myositis on the basis of weakly positive antibody tests for SRP and Mi‐2, leading to 2 years of treatment with high‐dose steroids and immunosuppressants. It was only through muscle biopsy and OGM that an SV in the *DMD* gene was identified, confirming the diagnosis of DMD. The chromosome karyotype test could not confirm this variation because the banding pattern was almost identical before and after the SV, making it indistinguishable. The ability of OGM to precisely locate the breakpoint was instrumental in establishing the clinical diagnosis as it uncovered a mutation that previously went undetected by other techniques. Similarly, Case 2 was diagnosed with congenital myopathy without muscle biopsy as the parents did not provide consent for biopsy. The patient's condition deteriorated until the age of 8 years, which is when OGM detected an SV in the *DMD* gene, leading to a definitive diagnosis of DMD and the initiation of regular treatment. The presence of this rare genetic variation, which was inherited from the patient's mother, highlights the importance of clear genetic identification for prenatal consultations and informed family planning. The investigation of these two cases revealed that SVs could be the underlying cause when clinical symptoms strongly suggest a diagnosis of DMD but conventional methods like MLPA and NGS yield inconclusive results. The precise detection capabilities of the OGM technology highlight its potential to prevent misdiagnosis or missed diagnosis, underscoring its importance in the clinical assessment of DMD and the broader context of genetic analysis.

Unbalanced translocations between the X chromosome and an autosome are uncommon, affecting about 1 in 30,000 newborns.^18^ This happens when two chromosomes break and swap their fragments, creating two new rearranged chromosomes. If a female has a breakpoint on her X chromosome that affects a gene that codes for a protein, she may suffer from disorders inherited in an X‐linked recessive manner (such as DMD).^19^ To avoid damage caused by abnormal genes on the autosomes, normal X chromosomes are usually (but not always) inactivated.^20^ The reported likelihood of patients with an X–autosome translocation showing a clinical phenotype is ~6%.^21^

A 2016 literature review of 43 female DMD carriers with X chromosome translocations revealed that chromosome 9 had the highest translocation frequency at 16%, followed by chromosomes 4 and 22, each with a translocation frequency of 11.3%.^22^ Depending on the size of the swapped fragment, reciprocal translocations can be identified by chromosome banding studies and/or fluorescence *in situ* hybridization (FISH).^23^ However, chromosome karyotyping and FISH techniques cannot easily identify whether the chromosome breakpoints involve the *DMD* gene. The OGM technology can detect small structural changes and locate the breakpoints more accurately.^24^

In this study, OGM pinpointed the balanced translocations between the X chromosome and autosomes for two female dystrophinopathy patients, clarifying the chromosomal rearrangement breakpoints and their gene and positional impacts. Case 5 showed a translocation between chromosomes 8 and X, which disrupted the *DMD* gene intron 42, exon 43, and intron 43 and caused mild symptoms and elevated CK levels. Case 6 showed a translocation between chromosomes 5 and X, which impacted the *DMD* gene intron 1 and manifested as developmental delay, muscle weakness, high CK levels, and pronounced DMD symptoms since childhood. Symptom severity in such female patients may be correlated with X chromosome inactivation patterns.^22^ The distinct *DMD* gene breakpoint locations could influence clinical symptom severity, warranting further investigation.

In this investigation, Cases 3 and 4, both confirmed DMD patients, exhibited non‐contiguous exon duplications or deletions on MLPA. Upon OGM reanalysis, the initial finding of the deletion of exon 42 in Case 4 was found to be inaccurate, and the exon was found to be present, suggesting that the deletion was either too small to be detected by OGM or that a point mutation was present in the MLPA probe for exon 42; the findings of Sanger sequencing supported the latter. The true mutation was a frameshift mutation caused by the deletion of exons 45–50. Case 3, after retesting with the OGM technology, was found to have two non‐contiguous exons duplicated, which was originally an SV. Such findings underscore the need to account for false‐positives or SVs when detecting non‐contiguous exon changes in the *DMD* gene, with OGM providing a definitive confirmation.

In the study of DMD genetics, recent molecular techniques have shown significantly improved diagnostic accuracy. MLPA is particularly good at detecting large‐scale changes in the *DMD* gene, such as deletions or duplications of exons. However, it may not identify smaller genetic variations, which may lead to a missed diagnosis.^4^, ^7^ Similarly, NGS can identify a wider range of genetic changes, including those in the exon and nearby intron regions; however, its efficacy can be limited if the probe does not cover the entire area.^4^ OGM has emerged as a valuable tool in *DMD* gene detection, offering high‐resolution detection of SVs, including insertions, deletions, inversions, duplications, and translocations.^25^ This method surpasses classical karyotyping and microarrays in its ability to identify complex and balanced structural changes without amplification bias. It stands out for its sensitivity to large SVs, improving upon the false‐positive and false‐negative rates associated with NGS.^14^

As early as 2017, Barseghyan et al. used OGM to accurately delineate large deletions, insertions, and translocation SVs in the *DMD* gene.^14^ In recent years, the integration of OGM into prenatal diagnostics has substantially evolved,^26^, ^27^ with emerging reports of its application in the detection of genetic disorders like *CDKL5* gene‐related conditions and facioscapulohumeral muscular dystrophy.^28^, ^29^ In 2023, OGM proved instrumental in the identification of SVs in a patient with a complex genetic diagnosis from a rare DMD subset.^25^ Despite these strides, the adoption of OGM in *DMD* gene detection is still not widespread.

In our investigation, OGM was used to accurately identify gene variants in six cases of patients in whom establishing genetic diagnoses for dystrophinopathy was previously faced with challenges. This study highlights the importance of complex SVs, which are often implicated in cases of dystrophinopathy that remain undiagnosed. OGM has proven to be a valuable method for unraveling complex SVs and identifying precise breakpoints in X–autosomal translocations, particularly in female dystrophinopathy patients. Our results advocate for the integration of OGM into the diagnostic process as it offers a significant advantage in detecting intricate genetic alterations that may otherwise go unnoticed.

In conclusion, OGM has technical advantages in identifying gene SVs and locating breakpoints precisely, and these benefits may increase its clinical adaptation, improve diagnostic sensitivity and accuracy, and assist clinicians in diagnosing challenging cases and providing early treatment to improve outcomes.

Our study has some limitations. First, two patients in our study yielded negative results, possibly due to deep intron mutations. Previous literature indicates that deep intron mutations comprise 0.6% of mutations in DMD patients^8^, ^30^ The clinical testing method used (whole‐exome sequencing) and the OGM technique used in our study cannot identify deep intron mutations. In upcoming investigations, we aim to further investigate mutations in these two patients using sequencing techniques that span the entire *DMD* gene. Second, the cohort size was small, comprising only eight individuals, with six showing positive outcomes through OGM. The limited sample size is attributable to the rarity of DMD and the scarcity of complex genetic cases, which pose challenges with obtaining a larger dataset. Consequently, further studies with larger cohorts are warranted to enhance our comprehension and validate our results.

## Conflict of Interest

No potential conflict of interest was reported by authors.

## Author Contributions

Jiahui Mai contributed to the methodology and writing the original draft. Jing Duan also worked on the methodology and writing the original draft. Xiaoyu Chen was responsible for data curation and visualization. Liqin Liu handled data curation and formal analysis. Dachao Liang focused on formal analysis and software development. Tao Fu contributed to formal analysis and visualization. Gang Lu was involved in investigation and validation. Wai Yee Chan provided supervision and contributed to the review and editing of the manuscript. Xufeng Luo was responsible for data curation. Feiqiu Wen secured funding and contributed to the review and editing of the manuscript. Jianxiang Liao was involved in funding acquisition and validation. Zhuo Li handled conceptualization and project administration and contributed to the review and editing of the manuscript. Xinguo Lu was responsible for conceptualization and project administration.

## Data Availability

The data that support the findings of this study are available on request from the corresponding author. The data are not publicly available due to privacy restrictions.
